# Effects of borax loading on the self-healing properties of epoxidized natural rubber†

**DOI:** 10.1039/d5ra00773a

**Published:** 2025-04-17

**Authors:** Tamonwan Chantaramanee, Supachok Tanpichai, Anyaporn Boonmahitthisud

**Affiliations:** a Department of Materials Science, Faculty of Science, Chulalongkorn University Bangkok 10330 Thailand anyaporn.b@chula.ac.th; b Learning Institute, King Mongkut's University of Technology Thonburi (KMUTT) 126, Pracha Uthit Road, Bangmod, Thung Khru Bangkok 10140 Thailand supachok.tan@kmutt.ac.th; c Cellulose and Bio-based Nanomaterials Research Group, King Mongkut's University of Technology Thonburi (KMUTT) 126, Pracha Uthit Road, Bangmod, Thung Khru Bangkok 10140 Thailand; d Center of Excellence in Green Materials for Industrial Application, Faculty of Science, Chulalongkorn University Bangkok 10330 Thailand; e Upcycled Materials from Industrial and Agricultural Wastes Research Unit, Department of Materials Science, Faculty of Science, Chulalongkorn University Bangkok 10330 Thailand

## Abstract

A promising approach for developing self-healing polymer materials involves the formation of reversible dynamic crosslinked networks. However, most self-healing systems require external stimuli such as temperature or pressure, to achieve effective healing. In this study, we successfully developed self-healing epoxidized natural rubber (ENR) materials that do not depend on external stimuli by incorporating borax as a crosslinking agent. The results demonstrated that borax facilitates self-healing efficiency through the reversibility of borate–ester and hydrogen bonds. ENR with 10 phr borax exhibits remarkable self-healing performance, achieving 94% efficiency in tensile strength and 95% efficiency in elongation at break after healing at ambient temperature for 24 h. Moreover, the effect of borax loadings on the chemical structure, thermal stability, and mechanical properties of the crosslinked ENR materials were investigated. These findings highlight the crucial role of borax in imparting self-healing properties to ENR without requiring external stimuli, offering an effective approach for developing self-healing elastomers.

## Abstract 2

Self-healing rubber without the need for external stimuli was fabricated by incorporating borax into epoxidized natural rubber (ENR) under alkaline conditions.

## Graphical abstract

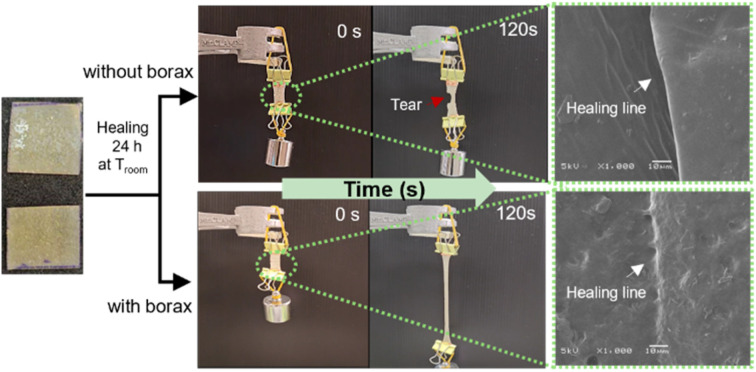

## Introduction

The increasing production and consumption of polymer-based materials have raised severe environmental concerns due to their non-biodegradable nature and inadequate waste disposal such as landfilling and incineration.^1^ Therefore, innovative solutions are required to reduce polymer waste and extend the lifespan of polymers. Moreover, the concept of a circular economy emphasizes waste minimization, reuse, and recycling of polymer products, promoting more sustainable material management.^2^ Accordingly, self-healing polymeric materials have become considerably attractive due to their ability to autonomously repair damage and restore their original properties and functionality.^3^ These materials offer a promising strategy for sustainability by enhancing the reliability and durability of polymer-based products, while extending their lifespan.

Based on their self-healing mechanisms, self-healing polymeric materials can be categorized into two main types: extrinsic and intrinsic healing. Extrinsic healing involves the incorporation of microcapsules or microvascular structures filled with healing agents that are released when damage occurs.^4^ However, this approach is limited by the finite supply of healing agents, which restricts the number of possible healing cycles. In contrast, intrinsic healing relies on the introduction of reversible dynamic bonds, such as hydrogen bonds, ionic bonds, aliphatic disulfide bonds, Diels–Alder, and *trans*-ester bonds.^5–11^ These dynamic bonds can rearrange under ambient or specific conditions, enabling effective healing while preserving or even improving the mechanical properties of the polymers.^12^ In recent years, intrinsic self-healing approaches have gained significant research interest due to their ability to autonomously repair damage without requiring external healing agents and their ability for multiple healing cycles.^13,14^ This makes intrinsic self-healing a promising solution for sustainable and long-lasting material applications. The dynamic bonds can be incorporated into self-healing polymeric materials by modifying polymer backbones, introducing dynamic crosslinkers, or adding functional fillers. These strategies enhance the material's ability to form the dynamic bonds at interfaces of polymer–polymer, polymer–filler, and filler–filler, thereby improving self-healing efficiency and mechanical performance.^15,16^ For example, Liu *et al.* modified polybutadiene with acetoacetyl groups (PBAA) and subsequently crosslinked PBAA with diamines to form vinylogous urethane dynamic bonds. The crosslinked PBAA was reprocessed under 10 MPa at 150 °C for 30 min.^17^ Also, Cui *et al.* incorporated extracted lignin into diglycidyl ether terminated polyethylene glycol (PEG) in the melt phase. The interaction between lignin and PEG resulted in a crosslinked network of both covalent and hydrogen bonds. The hydrogen bond networks provided rapid self-healing, achieving ∼90% recovery within 1 h at ambient temperature.^18^

Epoxidized natural rubber (ENR) is a derivative of natural rubber (NR), where epoxide groups are introduced randomly along the rubber chains *via in situ* epoxidation using formic acid and hydrogen peroxide.^19^ The introduction of epoxide rings enriches ENR with enhanced properties, such as solvent and oil resistance, superior wipe grip, gas impermeability, and low rolling resistance.^19–21^ Notably, under acidic conditions and high temperatures, ENR undergoes a ring-opening reaction, leading to the formation of diols, hydroxyl groups, and carbonyl groups in the ENR molecular chains.^22^ These functional groups contribute to the self-healing properties of ENR through polar interactions.^23,24^ Moreover, these oxygen-containing groups can further enhance the self-healing properties of ENR through interactions with other functional groups. For example, Feng *et al.*^7^ developed dodecanoic acid-crosslinked ENR supplemented with a small amount of oligoaniline. This material exhibited self-healing properties with a healing efficiency of 80% after undergoing trans-esterification treatment at 200 °C for 30 min. Furthermore, Xu *et al.*^25^ synthesized ENR composites with citric acid-modified bentonite, achieving restoration of tensile strength and elongation at break to 94% and 96%, respectively, after healing at 150 °C for 3 h. However, achieving sufficient healing properties often necessitates high healing temperatures, which can lead to adverse effects such as thermal degradation, excessive crosslinking, and an increase in glass transition temperature (*T*_g_) of rubber materials. These factors may negatively impact on the mechanical properties, healing performance, and overall service life of the materials.^26,27^

Borax is well-known for its role in crosslinking diol-containing polymers during hydrogel fabrication.^28–30^ Researchers have reported the efficacy of borax as a crosslinker in hydrogels, endowing them with their self-healing properties without the need for external stimuli. For example, Wang *et al.* utilized borax as a crosslinker to prepare polyvinyl alcohol (PVA) hydrogel through borate–ester and hydrogen bonds, imparting excellent mechanical properties. The compressive strength of the PVA hydrogels increased from 2.1 kPa with 2 wt% of borax to 5.1 kPa with 5 wt% of borax. The hydrogels also exhibited reversible sol–gel conversion characteristic, highlighting the dynamic nature of borate–ester and hydrogen bonds, as well as their self-healing properties.^31^ This strategy has been widely used to develop self-healing hydrogels. Yan *et al.* fabricated dual networks of the PVA, sodium alginate (SA), and borax hydrogels. The synergetic reversible interactions, including borate–ester and hydrogen bonds between PVA, SA, and borax, act as the energy dissipated centre, enhancing stretchability, toughness and providing rapid self-healing properties at ambient temperature.^32^ Moreover, Farajpour *et al.* reported that the incorporation of borax into ethylene–propylene–diene terpolymer/Kavlar/carbon fiber composites not only improved mechanical properties and abrasion resistance but also enhanced heat insulator properties.^33^ Similarly, Intharapat *et al.* fabricated boric acid-supported NR through a simple reaction between boric acid and NR containing hydroxyl groups. These hydroxyl groups were obtained *via* the ring-opening reaction of oxirane rings in ENR. The study revealed that the incorporation of boric acid enhanced thermal resistance and flame retardancy of the rubber materials.^34^ Our previous research has pioneered the incorporation of borax into ENR.^35^ We found that pH regulations considerably influenced the effect of borax on ENR properties. When prepared under neutral conditions (pH 7), most of the borax was transformed into boric acid (B(OH)_3_) within ENR/borax films, hampering the effective promotion of crosslinked network formation within ENR. Conversely, under alkaline conditions (pH 11), most of the borax turned into borate anions (B(OH)_4_^−^), acting as a crosslinker capable of establishing borate–ester and hydrogen bonds with the diol groups of ENR. Moreover, the ENR/borax films preliminary exhibited self-healing properties owing to the dynamic nature of both bonds. However, there is limited evidence regarding the interactions between ENR and borax, including mechanical properties and self-healing performances.^35^

Herein, we synthesized ENR under alkaline conditions (pH 11) using borax as a crosslinker, as at this pH, the majority of borax complex transforms into borate anions (B(OH)_4_^−^), enhancing crosslinking efficiency. This study investigated the effect of varying borax loadings (0–30 parts per hundred of rubber, phr) on the crosslinked network, thermal properties, mechanical properties, and self-healing properties of ENR. Additionally, we examined the influence of healing time and healing temperature on the healing efficiency of the ENR materials to determine the optimum conditions for achieving superior self-healing properties.

## Experimental

### Materials

High ammonia-concentrated NR latex with 60 wt% dry rubber content and terric acid (16A-16, 10 wt%) were supplied from the Rubber Research Institute of Thailand (Bangkok, Thailand). Formic acid (98 wt%), hydrogen peroxide (35 wt%), ammonia hydroxide (NH_4_OH) (30 wt%), and borax were purchased from Fisher Chemical Co., Ltd (Thailand), Asian Scientific Co., Ltd (Thailand), Panreac Química SLU, (Spain), and Qchemical Co., Ltd, (Thailand), respectively.

### Preparation of ENR with borax

ENR with ∼22 mol% epoxide and ∼2 mol% hydroxyl was synthesized *via in situ* epoxidation using formic acid and hydrogen peroxide, following our established procedure.^35^ The epoxide content and hydroxyl content were calculated from H-NMR spectra using the following equations:1
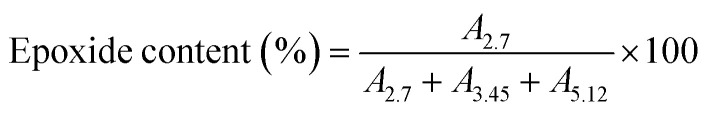
2
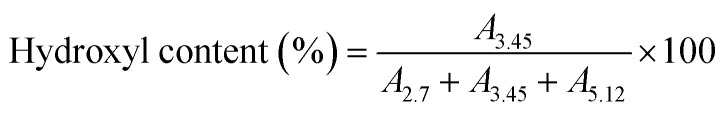
where *A*_2.7_, *A*_3.45_ and *A*_5.12_ are characteristic peak integrals of the peaks at 2.7 ppm (–C–O–C– of oxirane ring in ENR), 3.45 (C–OH of diols or hydroxyl-ester, obtained from oxirane ring-opening reaction), and 5.14 ppm (C=C of isoprene unit in ENR), respectively.^35^

In brief, the NR latex was diluted to 20 wt% before the addition of terric acid, which was used to stabilize NR to prevent the coagulation during epoxidation. The latex was then mixed with formic acid and hydrogen peroxide at a specific temperature, maintaining a ratio of formic acid to hydrogen peroxide per mole of isoprene of 0.75 : 1. Subsequently, the pH of the ENR latex was adjusted to 11 using NH_4_OH. A borax solution with various concentrations of 0, 5, 10, 20, and 30 phr, equivalent to 0, 4.76, 9.09, 16.67, 23.08 wt%, respectively, was gradually added to the ENR latex. The mixture was continuously stirred and allowed to react overnight at room temperature. The stirred ENR/borax latex was then cast into a mold and dried at 60 °C in an oven for 48 h. Afterward, the dried ENR/borax films were immersed in distilled water for 30 min to eliminate any excess chemicals deposited on the film surfaces. Finally, the samples were dried in the oven for 24 h at 40 °C. All samples were coded with *n*B, where *n* indicated the borax concentration (phr) applied in ENR films.

### Characterizations

The Fourier-transform infrared (FTIR) spectra of the ENR/borax samples were obtained using a Thermo Scientific Nicolet iS50 FTIR spectrometer (Waltham, USA) equipped with an iD5 attenuated total reflectance accessory. Samples were scanned over the wavenumber range of 4000–500 cm^−1^ with a resolution of 4 cm^−1^. Additionally, the chemical structures of all ENR samples were analyzed using a Horiba Scientific LabRAM HR Evolution KH 8700 Raman spectrometer (Kyoto, Japan) equipped with a 785 nm laser source. Raman spectra were collected over the wavenumber range of 2000–500 cm^−1^ with an acquisition time of 10 s.

^11^B-solid-state NMR spectra of ENR incorporated with various loadings of borax were acquired with an Avance-III 400 NMR spectrometer (Bruker, USA) at 128 MHz frequency and a spinning rate of 8 kHz. The samples were filled in standard ZrO_2_ rotors with a diameter of 4 mm. Borax was used as an external reference.

Equilibrium swelling experiments were conducted to investigate the crosslink densities and gel contents of the ENR films with various borax contents. Initially, a rubber piece was immersed in toluene for 3 days. Subsequently, the rubber piece was removed from the toluene, wiped with tissue paper to remove excess solvent, and immediately weighed (*W*_1_) using an analytical balance. After that, the swollen rubber piece was dried in an oven at 40 °C until a constant weight (*W*_2_) was achieved. The measurements were performed in triplicates.

The crosslink density was calculated based on the Flory–Reihner equation:^6,36^3
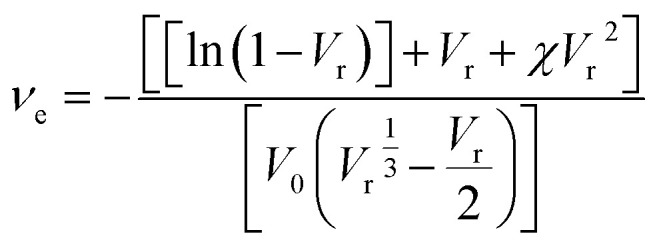
where4
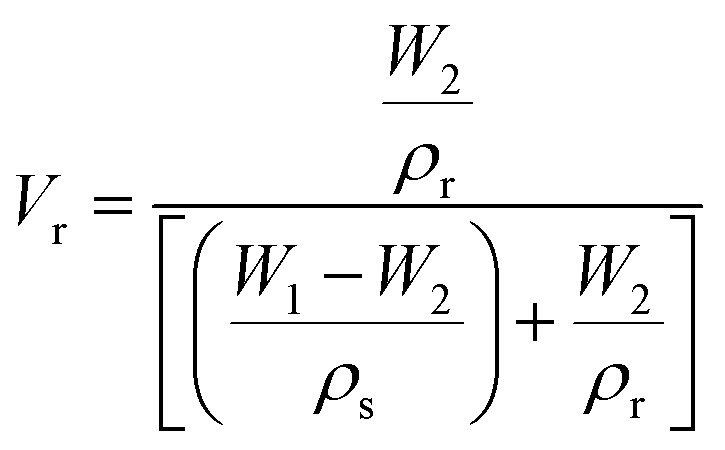
Here, *ν*_e_ is the crosslink density (mol cm^−3^), *V*_0_ is the molar volume of toluene (106.2 cm^3^ mol^−1^), and *V*_r_ is the molar volume of rubber (cm^3^ mol^−1^). *χ* represents the Flory–Huggin's interaction parameter between ENR and toluene (*χ* = 0.39), while *ρ*_s_ and *ρ*_r_ are the densities of toluene (*ρ*_s_ = 0.865 g cm^−3^) and ENR (*ρ*_r_ = 0.96 g cm^−3^), respectively.^6,36^

The gel content was determined using the following equation:5
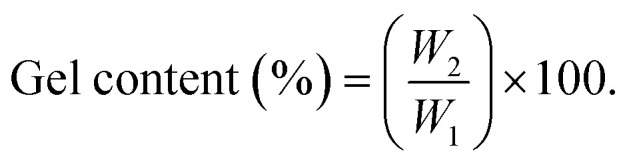


The thermal properties of the ENR/borax samples were assessed using differential scanning calorimetry (DSC) with a Mettler Toledo STARe DSC 822^e^ (Greifensee, Switzerland). First, samples of ∼5 mg were packed into aluminium pans, and the measurements were conducted from −60 °C to 20 °C, cooled to −60 °C and then heated to 20 °C again at a heating rate of 10 °C min^−1^ under a nitrogen atmosphere with flow rate 50 mL min^−1^. *T*_g_ was determined from the second heating cycle. Moreover, thermogravimetric analysis (TGA) was performed using a Metter Toledo TGA/SDTA 851^e^ (Greifensee, Switzerland) analyzer to investigate the thermal stability of the ENR/borax materials. Samples of ∼10 mg were heated under a nitrogen atmosphere with flow rate 20 mL min^−1^ from 50 °C to 100 °C at a heating rate of 20 °C min^−1^. The samples were then held at 100 °C for 20 min to eliminate absorbed water molecules before being heated again from 100 °C to 600 °C at a heating rate of 10 °C min^−1^.

The mechanical properties including tensile strength, elongation at break, modulus at 100% strain and tensile energy (the area under the stress–strain curves) of the ENR/borax materials were evaluated using a Lloyd LF-Plus universal testing machine (Berwyn, USA) equipped with a 50 N load cell. Dumbbell-shaped samples (4 mm × 75 mm × 0.4 mm) were prepared following ISO 37 standards and tested with a strain rate of 700 mm min^−1^ and a gauge length of 15 mm. Furthermore, the tensile fractural surface morphology was examined using field-emission scanning electron microscopy (FE-SEM) with a JEOL JSM-7610F, Oxford X-Max 20 (Tokyo, Japan), operating at an accelerating voltage of 5 kV. Before observation, the samples were coated with a thin layer of gold.

For the self-healing test, the sample was bisected using a sharp blade. Subsequently, two freshly cut surfaces were autonomously aligned using hands for 1 min and then pressed with a 100 g weight for another 1 min. The rubber pieces were left to heal at room temperature (30 °C) for 24 h. Following the healing process, the samples underwent tensile testing using the same testing condition as previously explained. The healing efficiencies (*η*) of the ENR/borax materials were determined by calculating the ratio of the mechanical properties of the healed samples to those of the original sample using the following formula:6
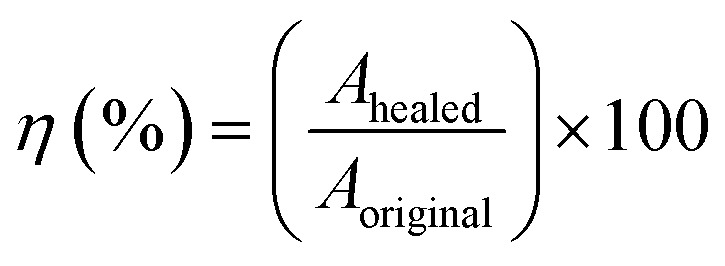
where *A*_original_ and *A*_healed_ are the mechanical properties (tensile strength, elongation at break, modulus at 100% strain, and tensile energy) of the original sample and the healed sample, respectively. Furthermore, the healing position was examined using SEM with a JEOL JSM-550 (Tokyo, Japan). The operating conditions and sample preparation are similar to those used in FE-SEM.

For statistical analysis, the experimental data were compared using one-way analysis of variance (ANOVA) with Tukey's HSD *post hoc* test and the *t*-test method using IBM SPSS statistic software. The statistical significance was considered at *p* < 0.05.

## Results and discussion

### Preparation and characterization of ENR/borax films

**Scheme 1:**
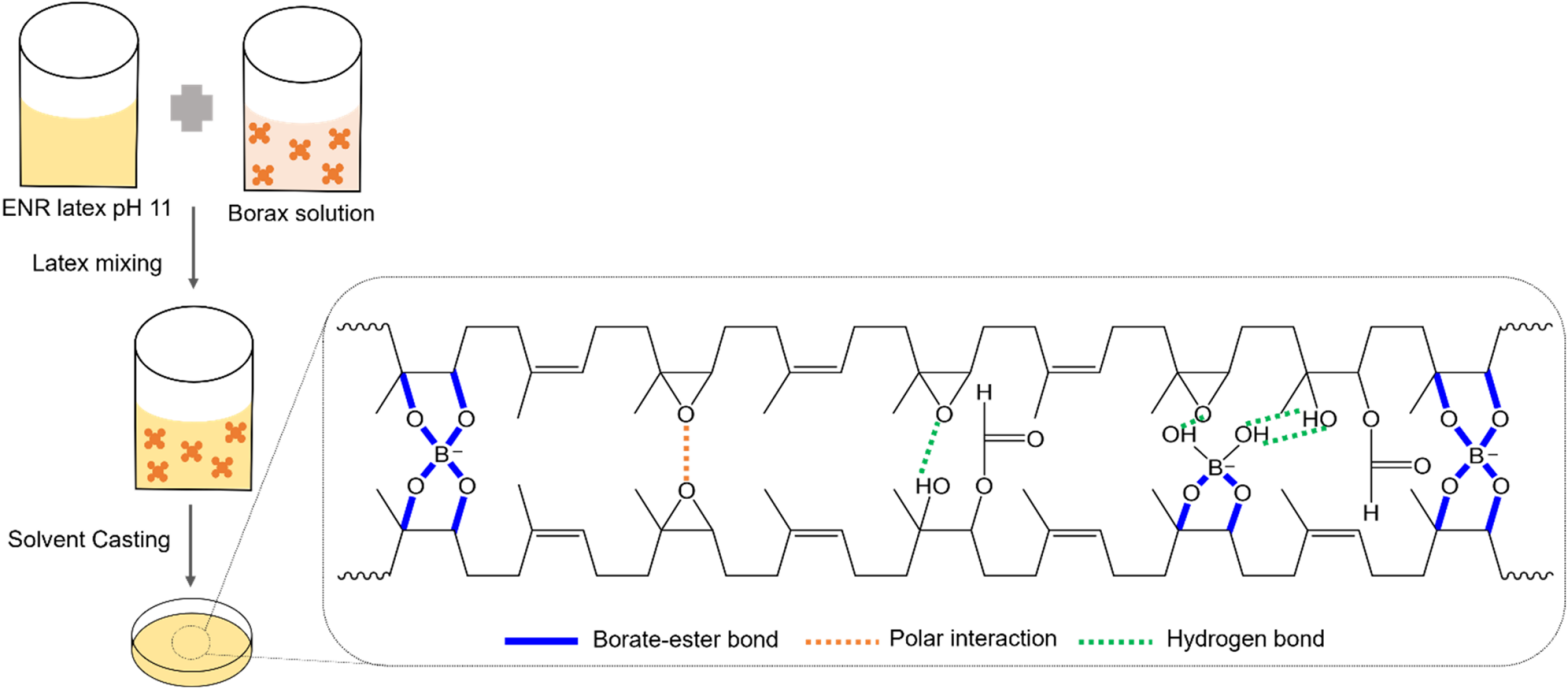
Proposed bonding mechanisms within the ENR/borax films.

The possible bonding mechanisms within the ENR/borax films are proposed in Scheme 1. Epoxide, diol, hydroxyl, and carbonyl groups were randomly introduced along the rubber chains through epoxidation and ring-opening side reactions.^22,37^ The hydrolysis of borax generated acid–based pairs of boric acid and borate anions, which could form borate–ester bonds with the diol functional groups of polymer chains.^38^ The diol complexation of borate anions occurred in two steps: first, the complexation between the diols of the polymer and adjacent hydroxyl groups of borate anions to form mono-chelate (L-B), followed by the formation of tetragonal bis chelate (L-B-L) with other diol groups of polymer chains.^39^ Our previous research found that the alkaline condition (pH 11) facilitated the formation of B(OH)_4_^−^, crucial in the diol complexation between ENR and borax. The results showed that alkaline conditions could more effectively promote a crosslinked reaction between ENR and borax compared to neutral conditions (pH 7).^35^ It should be noted that the acidic condition could facilitate epoxidation and ring opening reaction during drying process, resulting in uncontrolled of epoxide and hydroxyl content.^22^ Thus, acidic condition does not suitable for synthesis ENR/borax films.

**Fig. 1:**
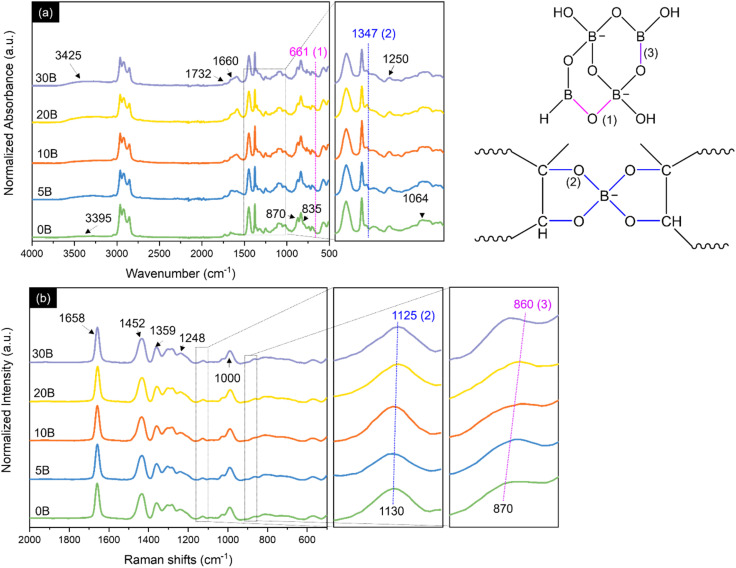
(a) FTIR spectra and (b) Raman spectra of neat ENR and ENR/borax films.

**Fig. 2:**
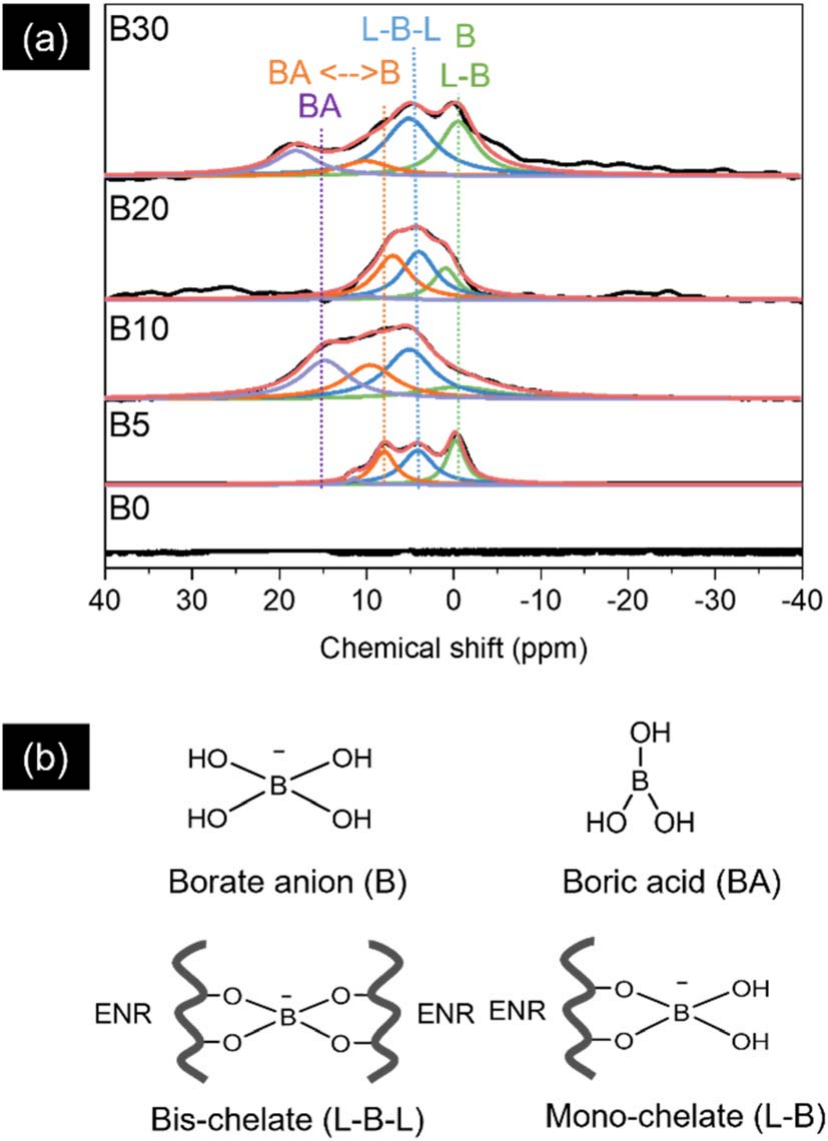
(a) ^11^B-NMR spectra and deconvoluted spectra using the Lorentz function and (b) schematic structure of boron species.

Fig. 1(a) shows FTIR spectra of the neat ENR and the ENR/borax films. The absorption peak located at 835 and 1660 cm^−1^ corresponds to C=C vibration and C=C stretching of *cis*-1,4-polyisoprene, respectively. Moreover, the absorption peaks located at 870 and 1250 cm^−1^ are assigned to the asymmetric and symmetric stretching vibration of epoxide, respectively, confirming successful epoxidation.^40,41^ Furthermore, the peaks observed at 1064 and 1732 cm^−1^ and the broad peak appearing at 3395 cm^−1^ are attributed to C–O stretching of ester and aliphatic alcohol, C=O stretching, and OH stretching, respectively.^22,42,43^ These peaks were indicative of the ring-opening side reaction that may occur owing to the high temperature and acidity during the ENR synthesis.^22,37^ After adding borax, new peaks appeared at 661 and 1347 cm^−1^, corresponding to B–O–B bending and the asymmetric stretching of B–O–C, respectively, in the borate network.^44,45^ The hydroxyl peak (3395 cm^−1^) also shifted slightly to a higher wavenumber (3425 cm^−1^), indicating the formation of hydrogen bonding and borate–ester bonding between ENR and borate anions.^32,45^ The Raman spectra were further analyzed to confirm the interaction between ENR and borax, as shown in Fig. 1(b). Several characteristic peaks of ENR were also observed in Raman spectra, including 870 (C–O–C stretching), 1000 (C–CH_2_ stretching), 1130 (C–O), 1248 (C–O–C), 1359 and 1452 (CH_2_ deformation), and 1658 cm^−1^ (C=C stretching).^46–48^ With the presence of borax, the peak at 1130 cm^−1^ shifted to 1125 cm^−1^ and exhibited broadening, attributed to B–O–C bonding. Additionally, the emergence of a new peak at 860 cm^−1^ was assigned to the B–O stretching of B(OH)_4_^−^.^49,50^ Consequently, Boron complexation chemistry between ENR and borax was investigated by ^11^B-NMR. As shown in Fig. 2, B0 presented no signal of any boron complex due to the absence of borax. While the ENR samples with borax displayed 4 deconvoluted peaks as represented structure of boron species (Fig. 2(b)). The signal at 0 ppm corresponds to a mono-chelate complex (L-B) combined with free borate anion. The second peak at 5 ppm is a bis-chelate complex (L-B-L), which refers to a crosslinked form with borax. The third and fourth peaks at 10 ppm and 15 ppm are related to boron atom exchange between boric acid and borate anion and free boric acid.^51,52^ The presence of L-B and L-B-L confirmed the formation of borate–ester bonds between ENR and borax. Moreover, the degree of crosslinking was roughly estimated by the ratio of the area peaks (L-B-L/L-B), as reported in Table 1. It was found that the L-B-L/L-B ratio increased with an increase in the amount of borax until the amount of borax exceeded 10 phr. After that, the L-B-L/L-B ratio decreased. The decrease of the L-B-L/L-B ratio was attributed to an excessive amount of borax (in the form of borate anion) and an increase of L-B complex. It suggested that the suitable amount of borax as a crosslinker in this system was 10 phr. These findings confirmed not only the formation of the crosslink network between ENR and borate anions but also the existence of the borate network within the ENR.

**Table 1:** The area peak (%) of the deconvoluted B-NMR spectra and ratio of L-B-L/L-B

Samples	Area (%)				Ratio of L-B-L/L-B
	0	5	10	15	
B0	—	—	—	—	—
B5	27.66	43.03	27.49	1.82	1.56
B10	14.01	34.47	24.91	26.61	2.46
B20	19.15	40.28	37.98	2.59	2.10
B30	29.25	43.04	11.38	16.33	1.47

**Fig. 3:**
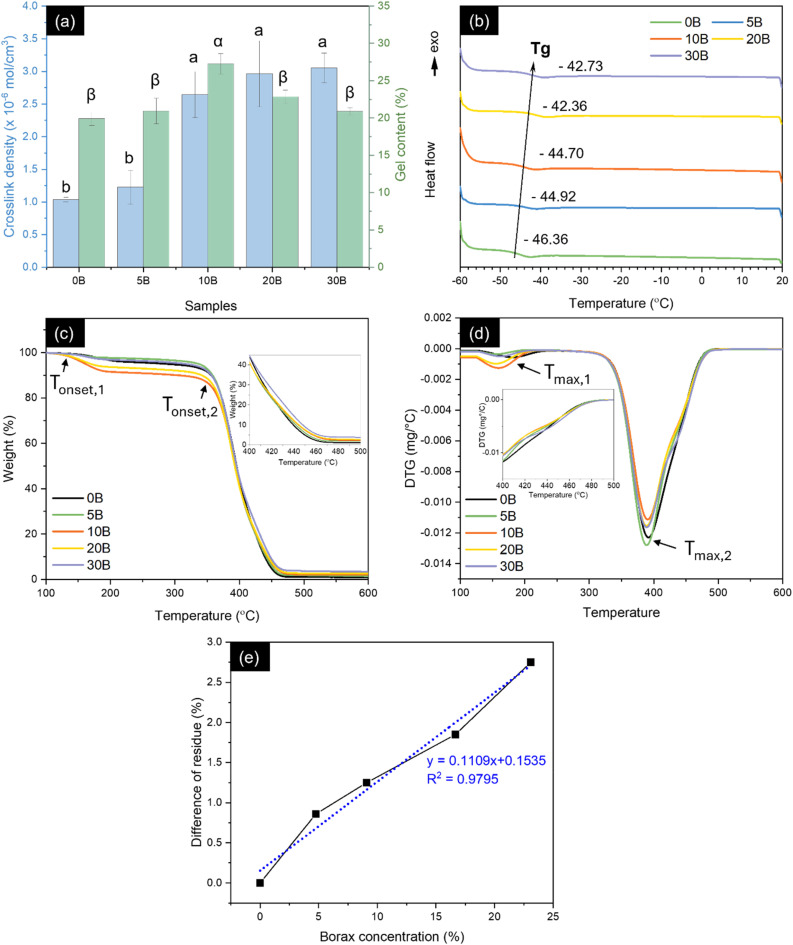
(a) Crosslink density and gel content, (b) DSC curves, (c) TGA curves, (d) DTG curves of the neat ENR and ENR/borax films and (e) relationship between the residue content of ENR/borax samples and the borax concentration (%). Superscripts (a, b, α and β) denote statistical significance (*p* < 0.05) among the tested samples, while *T*_onset_ and *T*_max_ abbreviate the onset degradation temperature and the maximum degradation temperature, respectively.

To further investigate the formation of crosslinking between ENR and borax, equilibrium swelling experiments and gel content analyses were conducted. As shown in Fig. 3(a), the crosslink density of 10B notably increased by 154%, from 1.04 × 10^−6^ to 2.65 × 10^−6^ mol cm^−3^. Beyond a borax loading of 10 phr, no significant change (*p* > 0.05) in crosslink density was observed. The gel content followed a similar trend to that of the crosslink density, showing a 37% increase for 10B compared to the neat ENR (0B). However, when borax content exceeded 10 phr, a decrease in gel content occurred, with reductions of 16% and 23% for 20B and 30B, respectively, compared to 10B. This decrease might be attributed to the presence of voids resulting from the washing of unreacted borax (B(OH)_4_^−^ and B(OH)_3_) and L-B complexes, as shown in Fig. S1,† allowing solvents to permeate and dissolve rubber chains. In Fig. 3(b), *T*_g_, as evaluated from the DSC thermograms of the prepared rubbers, gradually increased with increasing borax loadings. This suggested an enhanced restriction of chain mobility owing to the formation of a three-dimensional crosslinked network through borate–ester bonding and hydrogen bonding between ENR and borax, correlating with the increase in the crosslink density.^51^ These results confirmed the behavior of borax as a crosslinker in ENR/borax films.

### Thermal properties of ENR/borax films

The TGA and derivative thermogravimetric (DTG) curves, shown in Fig. 3(c and d), illustrate the thermal degradation behaviors of the ENR/borax materials, while Table 2 provides the onset degradation temperature (*T*_onset_), the maximum degradation temperature (*T*_max_), and residues (%) of the neat ENR and the ENR/borax films under a nitrogen atmosphere. The neat ENR (0B) undergoes a two-step thermal degradation process. Its initial thermal degradation, with a *T*_onset_ of ∼152 °C, was attributed to the removal of chemical residues from the epoxidation process used to prepare ENR. Subsequently, the main degradation state, corresponding to the degradation of the ENR chains, occurred with a *T*_onset_ of ∼368 °C.^34^ On the contrary, the crosslinked ENR exhibited thermal degradation in three distinct stages. The incorporation of borax led to an earlier onset of thermal degradation compared to the neat ENR, as evidenced by a decrease in *T*_onset,1_ by ∼15 °C and *T*_max,1_ by ∼20 °C. This phenomenon was attributed to the removal of bonded water within borax and the formation of boron oxide.^34,53^ The second degradation stage of the crosslinked ENR was similar to that of the neat ENR, indicating comparable degradation behaviour. However, a third degradation step was observed between 400 °C and 500 °C, as indicated by changes in the TGA slope and the shape of DTG curves. This degradation step could be attributed to the complexation between borax and ENR, as confirmed by FTIR, Raman and B-NMR spectra of the ENR/borax materials.

**Table 2:** Thermal degradation temperature and residues of the neat ENR and ENR/borax films^a^

Sample	*T*_onset,1_ (°C)	*T*_max,1_ (°C)	*T*_onset,2_ (°C)	*T*_max,2_ (°C)	Residues (%)
0B	151.65 ± 0.45	180.44 ± 1.89	368.46 ± 0.18	395.77 ± 0.45	0.82 ± 0.40
5B	134.08 ± 2.67	156.15 ± 0.49	367.62 ± 0.01	392.13 ± 0.04	1.68 ± 0.39
10B	133.45 ± 0.95	158.53 ± 1.34	367.80 ± 0.01	392.00 ± 1.12	2.07 ± 0.18
20B	132.47 ± 1.18	156.0 ± 0.74	366.09 ± 1.68	390.74 ± 0.88	2.67 ± 0.04
30B	142.37 ± 0.87	162.46 ± 0.03	366.95 ± 0.12	392.67 ± 0.22	3.57 ± 0.05

a Abbreviations: *T*_onset_, onset degradation temperature; *T*_max_, maximum degradation temperature.

The observed decrease in thermal stability of the crosslinked ENR, in comparison to the neat ENR, demonstrated that the presence of borax accelerated the thermal degradation of ENR. This observation indicated potential limitations in the application of the crosslinked ENR prepared in this study at temperatures exceeding 100 °C. Therefore, our future research will focus on incorporating reinforcing agents to enhance the thermal and mechanical properties of the crosslinked ENR materials for use at a higher temperature. Furthermore, the crosslinked ENR demonstrated higher char yield contents, compared to the neat ENR, with a linear relationship observed between the ENR/borax residue and borax concentration (%), as shown in Fig. 3(e). An increase in char residue as increasing borax concentration has also been reported in other studies using borax as crosslinker in polymer such as cellulose, guar gum, and starch/PVA blend.^30,54,55^

This increase in the char yield content, associated with a decrease in DTG peak height, could be ascribed to the combined effects of borax dehydration during degradation and the formation of a glassy coating layer of boron oxide on the carbonaceous residue of ENR. These mechanisms could effectively delay degradation by preventing the transfer of heat to the substrate and promoting an increase in char residues, indicating the flame-retardant properties of the ENR/borax films.^34^

### Mechanical properties of ENR/borax films

**Fig. 4:**
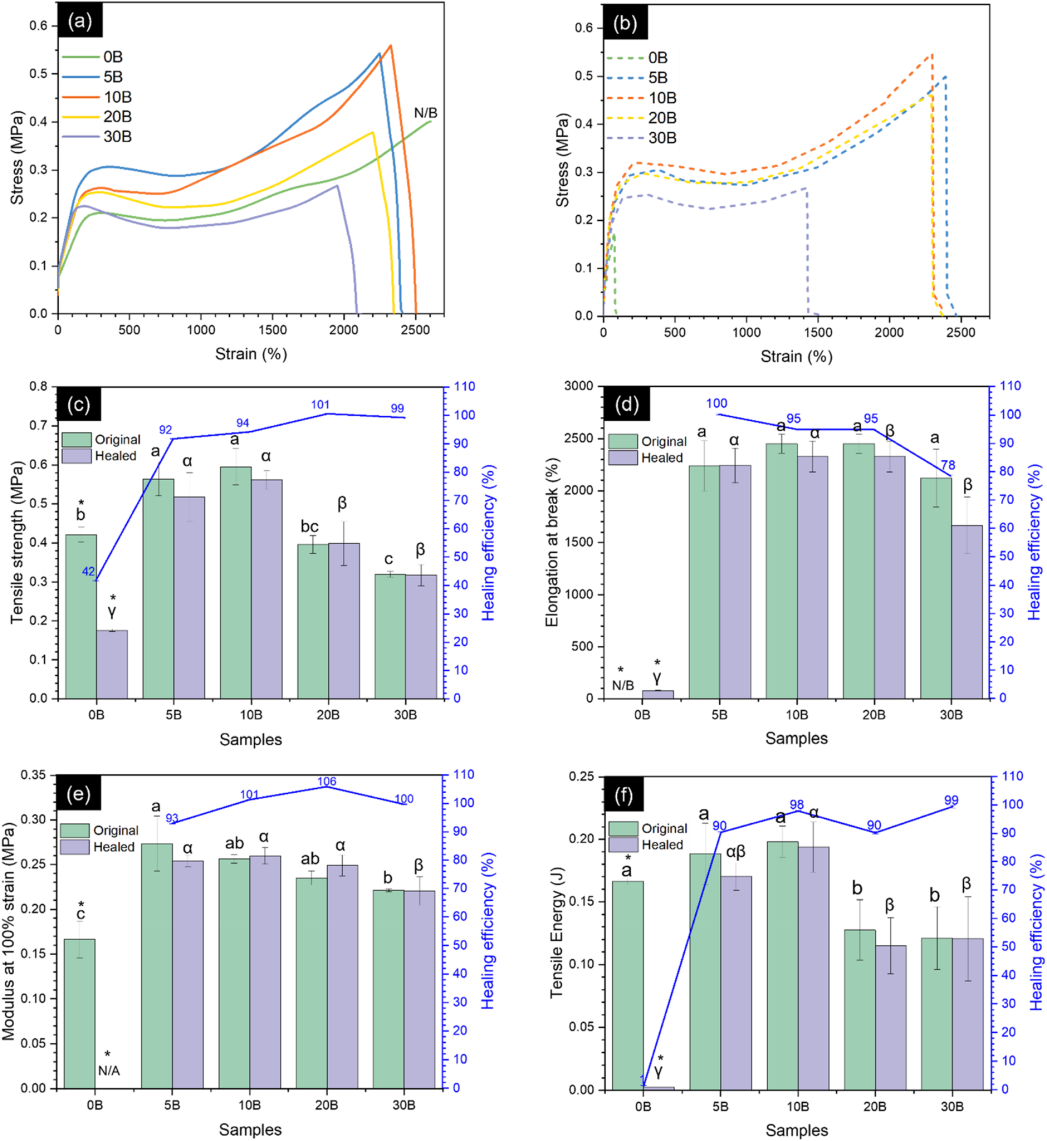
Stress–strain curves of (a) the original and (b) healed states of the neat ENR and ENR/borax films. (c) Tensile strength, (d) elongation at break, (e) modulus at 100%, and (f) tensile energy of the original ENR/borax films and after healing it for 24 h at room temperature (30 °C). Superscripts (a, b, α, and β) denote statistical significance obtained from one-way ANOVA and *post hoc* comparison (*p* < 0.05) among the tested samples. The symbolic (*) indicates significant difference between the original samples and healed samples using the *t*-test method. The term N/B refers to a sample that did not break.

The effect of borax acting as a crosslinker on the mechanical properties of ENR was evaluated through tensile testing at room temperature, and the stress–strain curves, along with the relevant tensile properties of the neat ENR and ENR/borax films, are shown in Fig. 4. The one-way ANOVA and *post hoc* comparison using Turkey's HSD demonstrated that adding borax significantly affected the mechanical properties of the ENR/borax films. Generally, the tensile strength, modulus at 100% strain, and tensile energy of ENR significantly increased with increasing borax content until reaching the maximum at 10 phr. Compared with the neat ENR (0B), the tensile strength, modulus at 100% strain, and tensile energy of 10B increased from 0.42 ± 0.02 to 0.59 ± 0.05 MPa (with an improvement of ∼43%), 0.17 ± 0.02 to 0.26 ± 0.005 MPa (with an improvement of ∼53%), and 0.17 ± 0.02 to 0.20 ± 0.00 J (with an improvement of ∼18%), respectively. However, a decline was observed in tensile strength, modulus at 100% strain, and tensile energy when the borax loading exceeded 10 phr (20B and 30B). This decrease could be ascribed to the excess borax remaining within the ENR matrix, which might act as defects similar to those found in the formation of agglomerated particles in ENR materials and the existence of pores/voids, as shown in Fig. S1.†^56,57^ Moreover, the presence of borax led to a decrease in elongation at break of the crosslinked ENR. The elongation at break of 10B slightly decreased to 2452 ± 91%, while 0B did not break even at the extension limit of the machine (2600% strain). The decrease in elongation at break was supported by the FE-SEM images of 0B and 10B, as shown in Fig. S2,† where 0B showed a ductile fracture, while 10B exhibited brittle fracture behavior. This behavior was attributed to the formation of the crosslinked network between ENR and borax, which restricted chain mobility of ENR, as evidenced by the increased crosslink density of the ENR/borax materials.

### Self-healing capabilities of ENR/borax films

The dynamic nature of the borate–ester and hydrogen bonding has been reported to enable the healing ability of polymer materials.^28,29,39^ Additionally, the presence of polar groups in ENR facilitates polar–polar interaction, serving as a driving force for chain diffusion.^23,32,58^ Moreover, the dynamic crosslinking of ENR facilitates the chain interdiffusion to the surface, thereby enhancing the self-healing ability of the materials.^59^ Herein, we further investigated the effect of borax contents, healing time, and healing temperature of the ENR/borax materials on their self-healing properties.

**Fig. 5:**
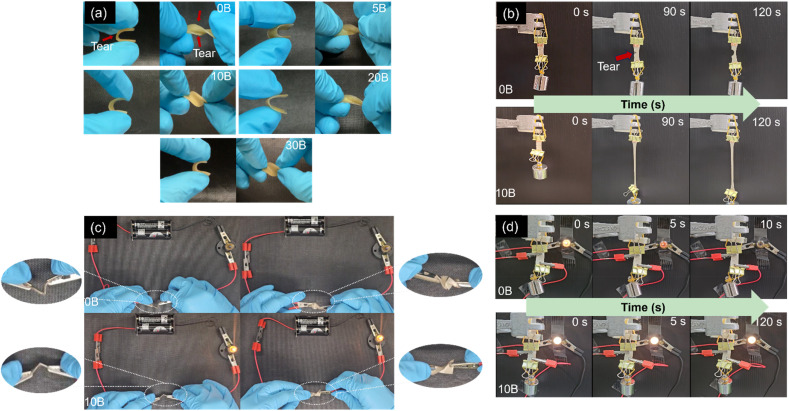
(a) Digital photographs of the healed 0B, 5B, 10B, 20B, and 30B samples under bending (left) and twisting (right). (b) Appearances of the healed 0B and 10B under tension (loading 100 g). Appearances of the healed 0B and 10B coated with silver paint connected to a circuit with a light bulb under (c) bending (left), twisting (right), and (d) under tension with a load of 50 g.

To investigate the effect of borax contents, the samples underwent a 24-hour healing period at room temperature (30 °C). The healing properties of the ENR materials were subsequently evaluated through tensile and macroscopic tests, as demonstrated in Fig. 4 and 5. Tensile tests were performed on both the original and healed ENR/borax films to quantify the healing efficiency. In the absence of borax, the mechanical properties of the healed sample were significantly low comparing to the original one. This was owing to the absence of the dynamic bonding. Specifically, the healing efficiencies based on tensile strength and tensile energy of 0B were 41.78 ± 0.06% and 1.45 ± 0.07%, respectively. On the other hand, upon the addition of borax, the mechanical properties of the healed sample did not show any significant difference compared to the original samples. This indicated the remarkable self-healing ability of the crosslinked ENR with borax, which could restore its mechanical properties. This effect was attributed to the high chain mobility resulting from the low *T*_g_ and synergetic effect between borate–ester and hydrogen bonds to effectively reconstruct its dynamic crosslinked network. The healing efficiencies of all mechanical properties increased dramatically to over 90%. Notably, the healing efficiency was calculated based on their original mechanical properties. While the original mechanical properties of 20B and 30B were slightly lower than those of 10B owing to their pores/voids and excess borax, as aforementioned, the amount of bonds based on the crosslink density did not considerably differ from that of 10B. Consequently, the healing efficiencies of 20B and 30B were close to 10B. Moreover, the healing efficiencies of all mechanical properties, except for % elongation at break, of 5B were slightly lower than those of 10B. This could be attributed to the fewer dynamic bonds, as evidenced by the lower L-B-L/L-B ratio and crosslink density of 5B compared to 10B. The healing efficiencies of tensile strength, elongation at break, modulus at 100% strain, and tensile energy of 10B increased to 94.25 ± 0.12%, 95.00 ± 0.10%, 101.31 ± 0.05%, and 97.95 ± 0.17%, respectively, compared to 0B. Additionally, the healing efficiency of the elongation at break of 30B gradually decreased, which might be attributed to the restriction of chain movement resulting from the formation of a crosslinked network between ENR and borax.

**Fig. 6:**
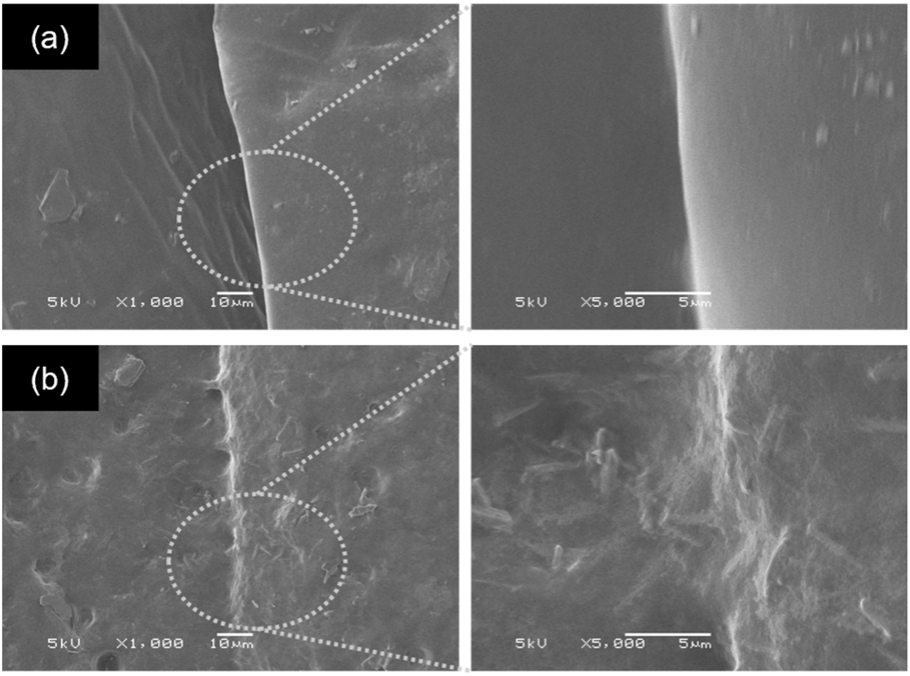
SEM images of the healing areas in (a) 0B and (b) 10B after healing at room temperature for 24 h.

The macroscopic results are illustrated in Fig. 5. The ENR/borax films displayed remarkable resilience, exhibiting no signs of fracture during bending and twisting. The 10B sample tolerated a loading of 100 g for over 120 s without showing any signs of fracture. In contrast, the uncrosslinked 0B sample clearly exhibited fractures at the joint section after healing under the same condition (Fig. 5(a and b)). Moreover, to delve deeper into the self-healing performance, the ENR/borax samples were coated with silver paint and connected to a light bulb circuit, as shown in Fig. 5(c and d). When subjected to bending and twisting, the bulb connected to 0B did not light up, and upon stretching with a 50 g mass, the bulb slowly faded over time, extinguishing completely after 15 s owing to a small tear at the joint fraction of 0B. Conversely, the bulb connected to the silver-coated 10B remained illuminated even when subjected to bending or twisting and continued to shine for over 120 s under stretching with a 50 g load. Furthermore, the healing areas of 0B and 10B after a 24-hour healing time were monitored, as presented in Fig. 6. There was no indication of healing at the healing position of 0B, whereas the two fracture surfaces of 10B exhibited well-fused characteristics, indicating the interdiffusion of ENR chains and the reconstruction of dynamic bonds at their interfaces. Considering the mechanical properties and self-healing efficiencies of 10B, the borax concentration of 10 phr represented an optimal condition for preparing crosslinked ENR materials. Notably, their observed healing efficiency exceeding 100% could be attributed to the low crosslink density and dynamic crosslink network, which allowed the rubber chains to move and entangle easily at fracture, facilitating the rearrangement of the dynamic bonds. Moreover, the effective chain mobility also induced the adjustment of the chain in the bulk into an optimal state.^60^

**Fig. 7:**
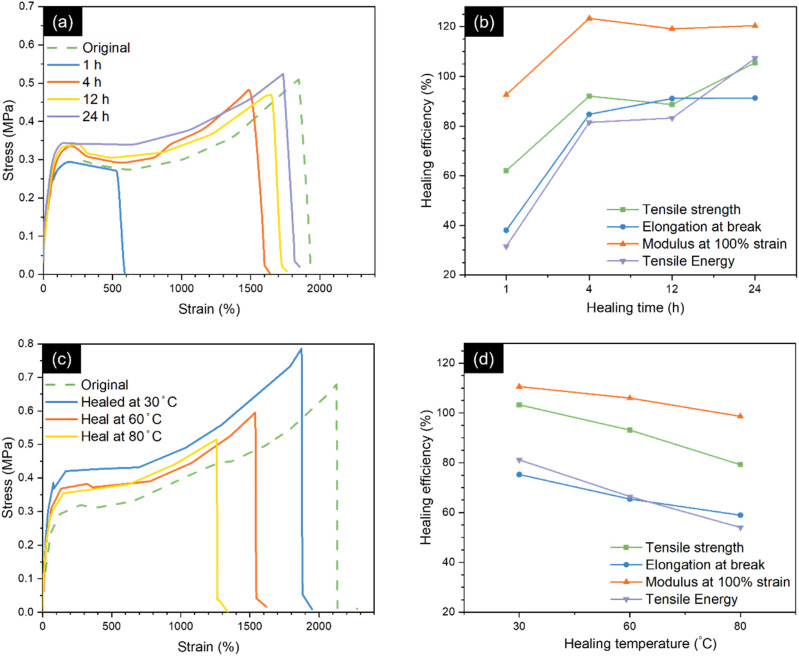
(a) Stress–strain curves and (b) healing efficiency of 10B after healing at 1, 4, 12, and 24 h at room temperature. (c) Stress–strain curves and (d) healing efficiencies of 10B after healing for 24 h at 30 °C (room temperature), 60 °C, and 80 °C.

The effects of healing times and healing temperatures on the healing efficiencies of 10B are presented in Fig. 7. The samples were healed at room temperature for various healing durations, including 1, 4, 12, and 24 h. Notably, after 1 h of healing, the healing efficiencies of tensile strength, elongation at break, modulus 100% strain, and tensile energy of 10B were 61.99 ± 0.21%, 38.02 ± 0.56%, 92.62 ± 0.14%, and 31.52 ± 0.50%, respectively. With an extended healing duration of 4 h, these healing efficiencies considerably increased to 92.02 ± 0.32%, 84.72 ± 0.36%, 123.36 ± 0.16%, and 81.46 ± 0.40%, respectively. The notable increase in the healing efficiencies of 10B with extended healing times aligned with previous findings.^8,61,62^ This could be attributed to the lower *T*_g_ of 10B compared to room temperature and the dynamic crosslink network between ENR and borax. These characteristics permit chain diffusion and interdiffusion of rubber molecules between cut surfaces, followed by the formation of dynamic bonds, including hydrogen and borate–ester bonds between ENR and borax, facilitating self-healing. Consequently, prolonged healing times allowed enhanced interdiffusion and provided ample time for the reconstruction dynamic bonding, leading to enhanced self-healing efficiency of 10B, evident in the healing efficiency exceeding 90% after 24 h of healing. Although extended healing durations could offer improved self-healing efficiency, the healing efficiencies at 4 hour of healing duration were higher than 80%. Which might be sufficient to yield ENR materials with mechanical properties similar to the original ones.

Furthermore, chain mobility can be promoted by thermal treatment. The effect of healing temperature on healing efficiency of 10B, with temperatures varied at 30 °C (room temperature), 60 °C, and 80 °C while maintaining a fixed healing time of 24 h was further investigated (Fig. 7(b)). Interestingly, increasing healing temperature reduced the healing efficiency of 10B. For example, the healing efficiencies based on tensile strength, elongation at break, modulus at 100% strain, and tensile energy of 10B were restored to 86.71 ± 0.13%, 63.98 ± 0.16%, 105.49 ± 0.13%, and 62.18 ± 0.11%, respectively, after healing at 60 °C. However, for 80 °C, the healing efficiencies were slightly decreased to 75.65 ± 0.21%, 58.91 ± 0.12%, 97.18 ± 0.12%, and 49.62 ± 0.13%, respectively. This reduction could be attributed to the reversible and exothermic reactions of the borate–ester bond between ENR and borax and the weakening of the hydrogen bonding at higher temperatures.^31,63,64^ Although higher chain mobility was found from thermal treatment, the reconstruction of hydrogen and borate–ester bonds might decrease with increasing temperature, resulting in a reduction of the self-healing ability. However, due to the *T*_g_ of the prepared ENR, which was lower than room temperature, along with the dynamic nature of borate–ester and hydrogen bonds, interdiffusion of rubber chains between the cut surfaces and the reconstruction of the dynamic bond networks occurred. This led to excellent self-healing properties without the need or external heat.

Table 3 compares the healing efficiencies and healing conditions of the crosslinked ENR obtained from this study and those of ENR with other crosslinkers. The crosslinked ENR materials with borax prepared in this study demonstrated excellent healing efficiency (exceeding 80%) after only 4 h of healing without requiring additional thermal energy. It is noteworthy that the thickness of the sample is a critical parameter influencing self-healing efficiency. In a prior study conducted by Yoon *et al.* the self-healing mechanisms of star polymers crosslinked with disulfide bonds by varying both the film thickness and the width of cut were examined. Their findings indicated that, with an increase in the initial thickness of the sample, larger cut could be effectively healed because surface tension was proportional to the exposed surface area, and the surface tension served as a driving force for the viscoelastic reflow of the exposed surfaces, facilitating their recontact to recovery damaged areas.^66^ The specimens of this work possessed a thinner dimension (0.4 mm) compared to those investigated in other studies (2 mm); however, the observed healing efficiency remains comparable to that of the thicker specimens. This finding suggested that borax could be a promising alternative as a crosslinker for fabricating rubber materials with superior healing properties at room temperature.

**Table 3:** Comparison of the healing efficiencies of ENRs prepared in this study and others^a^

Sample	Conventional crosslinker	Self-healing driving force	External stimuli conditions	Sample thickness (mm)	Healing efficiency (%)	Ref.
ENR/CABT (20 wt%)	—	Trans-esterification	150 °C, 3 h	0.5	TS ≈ 100, EB ≈ 100	25
ENR25/ZDMA (30 phr)	1 phr of DCP	Ionic bonds	80 °C, 30 min	1	TS ≈ 70, EB ≈ 76	6
ENR/CNCs (20 wt%)	—	Hydrogen bond supramolecular network	50 °C, 12 h	3	TS ≈ 95, EB ≈ 98	65
ENR/ZnO-CNF (5 phr)	0.5 phr of DCP and 1.6 phr of sulfur	Reversible ionic bond and hydrogen bond	80 °C, 1 h and room temperature, 3 h	0.4	TS ≈ 70, EB ≈ 92	36
ENR/CHI/A-CNC (2 phr)	—	Hydrogen bond supramolecular network	80 °C, 4 h	2	TS ≈ 90	61
ENR/borax (10 phr)	—	Borate–ester bond and hydrogen bond	Room temperature (30 °C), 4 h	0.4	TS ≈ 92, EB ≈ 85, TE ≈ 81	This work
ENR/borax (10 phr)	—	Borate–ester bond and hydrogen bond	Room temperature (30 °C), 24 h	0.4	TS ≈ 94, EB ≈ 95, TE ≈ 98	

a Abbreviations: TS, tensile strength; EB, elongation at break; TE, tensile energy; t-CNs, tunicate cellulose nanocrystals; CABT, citric acid-modified bentonite; ZDMA, zinc dimethacrylate; CNCs, chitin nanocrystals; ZnO-CNF, zinc oxide-modified cellulose nanofibers; CHI, chitosan; A-CNC, APTES-modified cellulose nanocrystals.

The mechanical properties of ENR/borax materials are relatively low due to the low crosslink density and the lack of a permanently crosslinked network. Xu *et al.* found similar phenomena in natural rubber (NR)/zinc dimethacrylate (ZDMA). They fabricated the self-healing NR by introducing an ionic crosslink network from ZDMA. Without the permanently crosslink network from DCP, the total crosslink density (ionic crosslink density) was around 0.33 × 10^−4^ mol cm^−3^ and tensile strength was relatively low (∼0.63 MPa). However, its superior self-healing was obtained after healing at 20 min at ambient temperature.^67^ Incorporating conventional crosslinker agents such as sulfur and peroxide in combination with borax, may offer a strategy to enhance the mechanical properties of ENR/borax materials. Notably, a higher concentration of permanently crosslinked networks could reduce chain mobility, decreasing the self-healing capability of rubber or requiring external stimuli to achieve promising self-healing performance.

Moreover, the incorporation of reinforcing materials can further improve mechanical properties. In the ENR/borax materials developed in this study, the presence of large polar groups, particularly the hydroxyl groups from borax and oxygen-containing groups in ENR, may improve compatibility between the matrix and polar filler such as cellulose, chitin, and chitosan. These fillers could form dynamic bonds (hydrogen and borate–ester bonds) with ENR and borax, potentially enhancing the mechanical properties of the ENR/borax materials while retaining their self-healing properties.^61,65,68^ Therefore, the development of self-healing materials with superior mechanical properties and higher crosslink density would be our future focus.

## Conclusions

The incorporation of borax in ENR induced a dynamic crosslinked network through borate–ester and hydrogen bonding. The borax incorporation significantly influenced the degree of crosslinking, mechanical properties and self-healing performance of the ENR/borax films. The optimum concentration of borax was determined to be 10 phr. The dynamic nature of borate–ester and hydrogen bonds allowed chain interdiffusion and bond network reconstruction after damage, resulting in remarkable self-healing properties of the ENR/borax films. Notably, for ENR with 10 phr of borax, their self-healing efficiencies in all mechanical properties were higher than 90% after healing at room temperature for 24 h. Furthermore, these films exhibited outstanding self-healing efficiency (>80%) after just 4 h of healing at room temperature. Extending the healing time slightly promoted the healing efficiency of the crosslinked ENR/borax materials whereas increasing the healing temperature hindered their self-healing ability. This study presents a promising strategy to fabricate a functional rubber material with the self-healing properties.

## Data availability

The raw/processed data required to reproduce these findings cannot be shared at this time as the data also forms part of an ongoing study.

## Author contributions

The manuscript was written through contributions of all authors.

## Conflicts of interest

There are no conflicts to declare.

## Supplementary Material

RA-015-D5RA00773A-s001
