# Supplementary material for: Effects of borax loading on the self-healing properties of epoxidized natural rubber
Source: RSC Adv. 2025 Apr 17;15(16):12087–99. doi: 10.1039/d5ra00773a (PMC12004222; doi:10.1039/d5ra00773a)
Supplement: RA-015-D5RA00773A-s001 [file RA-015-D5RA00773A-s001.pdf]

Supplementary Information (SI) for RSC Advances.
This journal is © The Royal Society of Chemistry 2025

Supporting information

Effects of borax loading on self-healing properties of epoxidized natural rubber

Tamonwan Chantaramanee [a], Supachok Tanpichai [*bc] Anyaporn Boonmahitthisud [*ade]

[a]Department of Materials Science, Faculty of Science, Chulalongkorn University, Bangkok, 10330, Thailand.

[b]Learning Institute, King Mongkut's University of Technology Thonburi (KMUTT), 126, Pracha Uthit road, Bangmod, Thung Khru, Bangkok, 10140, Thailand.

[c]Cellulose and Bio-based Nanomaterials Research Group, King Mongkut's University of Technology Thonburi (KMUTT), 126, Pracha Uthit road, Bangmod, Thung Khru, Bangkok, 10140, Thailand.

[d]Center of Excellence in Green Materials for Industrial Application, Faculty of Science, Chulalongkorn University, Bangkok, 10330, Thailand.

[e]Center of Excellence on Petrochemical and Materials Technology, Chulalongkorn University, Bangkok, 10330, Thailand.

* Email: anyaporn.b@chula.ac.th, supachok.tan@kmutt.ac.th

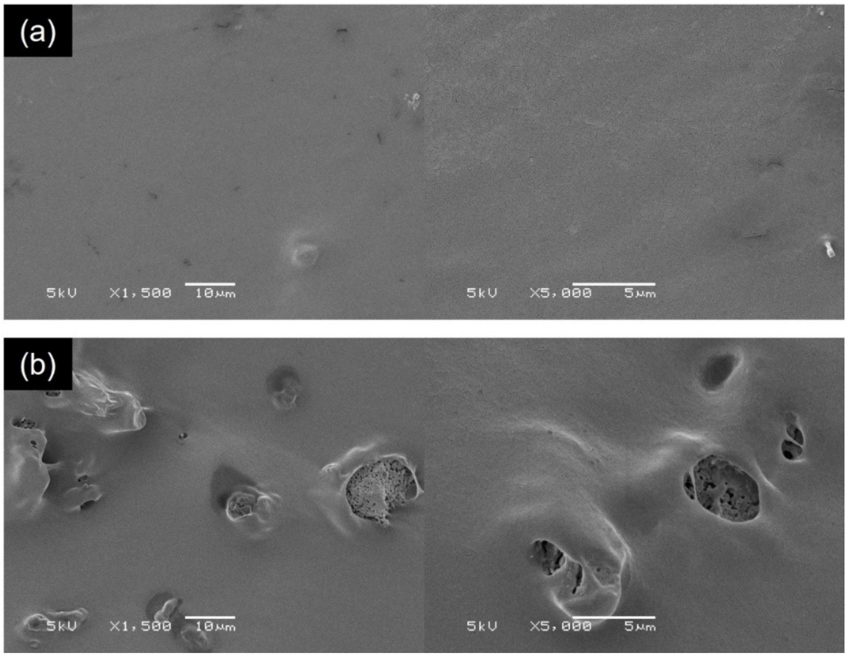

Figure S1. Cross-sectional surfaces of (a) 10B and (b) 30B materials.

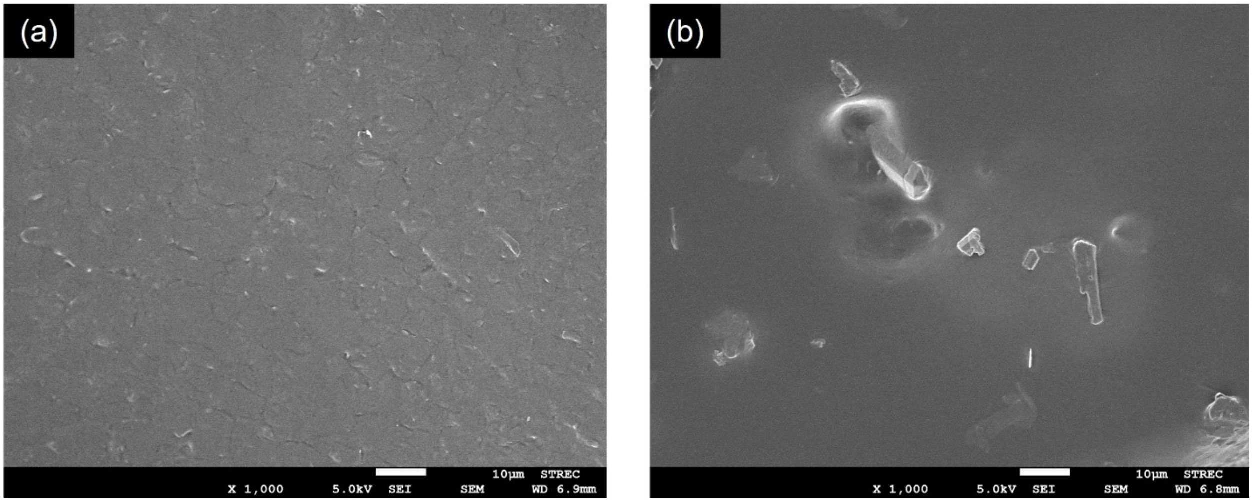

Figure S2. Fracture surface of the (a) 0B (b) 10B materials after tensile deformation.
